# The Role of Protein Kinase A Regulation of the E6 PDZ-Binding Domain during the Differentiation-Dependent Life Cycle of Human Papillomavirus Type 18

**DOI:** 10.1128/JVI.01234-13

**Published:** 2013-09

**Authors:** Craig P. Delury, Elizabeth K. Marsh, Claire D. James, Siaw Shi Boon, Lawrence Banks, Gillian L. Knight, Sally Roberts

**Affiliations:** School of Cancer Sciences, Cancer Research UK Cancer Centre, University of Birmingham, Birmingham, United Kingdoma; International Centre for Genetic Engineering and Biotechnology, Padriciano, Trieste, Italyb

## Abstract

Human papillomavirus (HPV) E6 proteins of high-risk alpha types target a select group of PSD95/DLG1/ZO1 (PDZ) domain-containing proteins by using a C-terminal PDZ-binding motif (PBM), an interaction that can be negatively regulated by phosphorylation of the E6 PBM by protein kinase A (PKA). Here, we have mutated the canonical PKA recognition motif that partially overlaps with the E6 PBM in the HPV18 genome (E6153PKA) and compared the effect of this mutation on the HPVl8 life cycle in primary keratinocytes with the wild-type genome and with a second mutant genome that lacks the E6 PBM (E6ΔPDZ). Loss of PKA recognition of E6 was associated with increased growth of the genome-containing cells relative to cells carrying the wild-type genome, and upon stratification, a more hyperplastic phenotype, with an increase in the number of S-phase competent cells in the upper suprabasal layers, while the opposite was seen with the E6ΔPDZ genome. Moreover, the growth of wild-type genome-containing cells was sensitive to changes in PKA activity, and these changes were associated with increased phosphorylation of the E6 PBM. In marked contrast to E6ΔPDZ genomes, the E6153PKA mutation exhibited no deleterious effects on viral genome amplification or expression of late proteins. Our data suggest that the E6 PBM function is differentially regulated by phosphorylation in the HPV18 life cycle. We speculate that perturbation of protein kinase signaling pathways could lead to changes in E6 PBM function, which in turn could have a bearing on tumor promotion and progression.

## INTRODUCTION

While in excess of 150 human papillomavirus (HPV) types have been identified, only a small subset has been defined as carcinogenic; these high-risk viruses cause cancers in the oropharynx, at sites on the skin, and at multiple sites in the anogenital tract, predominantly the uterine cervix (1–3). In squamous cell carcinoma of the cervix and oropharyngeal tumors, HPV16 is the most frequent type, whereas HPV18 is more strongly associated with cervical adenocarcinoma (4).

The infectious cycle of this small, double-stranded DNA virus is intimately linked to the terminal differentiation program of the host cell—the keratinocyte (reviewed in reference 5). The virus infects mitotically active keratinocytes within the basal cell layer of squamous epithelium, gaining access when these cells become exposed through microabrasions in the tissue. Here, the viral genome replicates as an episome, to levels of ∼50 to 200 copies per cell, and becomes segregated equally between daughter cells via synchronous replication with host DNA. Once infected cells migrate up from the basal cell layer, the normal occurrence of withdrawal of these cells from the cell cycle is prevented by the actions of the HPV early proteins E6 and E7. By working in cooperation, E6 and E7 establish a cellular milieu that supports amplification of the viral episome to thousands of copies per cell. Finally, expression of the viral capsid proteins and packaging of progeny virus are restricted to the more superficial differentiated cells.

The precise role of E6 in the virus life cycle is not fully understood, but the analysis of E6 mutants in the context of the complete viral genome has shown that E6 function makes a significant contribution to the productive life cycle (6–10). The ability of E6 to stimulate the degradation of p53, the tumor suppressor protein that is upregulated in response to E7 function in HPV-infected cells, is important for episomal maintenance in undifferentiated keratinocytes (8) and potentially for viral DNA amplification in suprabasal cells (10). However, other functions of E6 distinct from p53 degradation are also involved in supporting the virus life cycle (8, 10). One such E6 function is the ubiquitin-mediated proteolysis of a select group of PSD95/DLG/ZO-1 (PDZ) domain-containing proteins (referred to as PDZ proteins). The targeting of PDZ proteins by E6 is mediated by a C-terminal four-amino-acid class 1 PDZ binding motif (PBM) (X-S/T-X-V/L). Deletion of the E6 PBM in the context of the HPV16 and -31 genomes has been shown to interfere with stable maintenance of the viral genomes in keratinocytes, indicating that an intact E6 PBM is required for the normal E6 function in the infectious cycle (6, 7). To date, some 14 cellular PDZ domain-containing proteins have been shown to interact with E6. These include the human orthologues of the Drosophila melanogaster tumor suppressor proteins Discs Large (DLG1) and Scribble (hSCRIB) and the MAGUK with Inverted Domain (MAGI) proteins MAGI-1, -2, and -3 (11–15). These cellular proteins are primarily involved in the assembly of intercellular adhesion junctions, the regulation of cell polarity, proliferation, and invasion, and apoptotic processes (reviewed in reference 16).

Intriguingly, a canonical cyclic AMP (cAMP)-dependent protein kinase A (PKA) recognition motif (R-R/K-X-T/S) partially overlaps with the E6 PBM of several high-risk HPV types, with the critical amino acid at −3 of the PBM being the phosphoacceptor (17). From the analysis of crystal structures of the HPV18 E6 PBM bound to PDZ domains, phosphorylation of the threonine at −3 is predicted to disrupt binding to the PDZ domain through steric hindrance (18). Indeed, PKA phosphorylation of HPV18 E6 within the PBM has been shown to negatively regulate the interaction with DLG1 and MAGI-1, causing inhibition of DLG1 turnover by the proteasome, an effect that is dependent on the integrity of the PKA recognition motif within the C terminus of E6 (17, 19). Intriguingly, it has recently been shown that while phosphorylation of this domain blocks the interaction with PDZ domain-containing targets, it enables binding to a non-PDZ domain-containing protein, 14-3-3ζ, in a PKA-dependent manner (19).

Thus, the function of the E6 PBM may be differentially regulated by phosphorylation during the infectious cycle of the virus, not only to control interactions between E6 and PDZ domain-containing targets but also to control interactions with other host cell targets that only recognize the phosphorylated form of the E6 PBM. However, the biological relevance of regulation of this E6 domain in the context of the complete virus life cycle is not known. Therefore, to address this, we have constructed a mutation within the E6 gene of the HPV18 genome that disrupts the PKA recognition motif. The mutant genome was transfected into primary human foreskin keratinocytes (HFK) isolated from multiple donors and cell lines established. The effect of this E6 mutation on viral episome replication, maintenance, and amplification was examined and compared to HFK lines containing the wild-type (WT) HPV18 genome and to a mutant HPV18 genome coding for an E6 protein lacking the PBM. Our findings support the hypothesis that phosphorylation of the E6 PBM is an important mechanism for controlling E6 PBM function during the replication cycle of the virus.

## MATERIALS AND METHODS

### Construction of mutant HPV18 genomes and generation of stable cell lines.

A pGEMII plasmid containing the complete HPV18 genome inserted at the EcoRI restriction site at nucleotide 2440 (pGEMII18-WT; a gift from Frank Stubenrauch, University of Tübingen) was used as a template for site-directed mutagenesis (QuikChange; Stratagene). Two mutant genomes were generated: pGEMII18-E6ΔPDZ contains three nucleotide substitutions (G^567^→T, A^570^→T, and C^571^→G) within the E6 DNA sequence and inserts translation termination codons at amino acid positions 155 and 156. The primer set used was 5′-CGACTCCAACGACGCAGATAATGACAAGTATAATATTAAGTATG-3′ (forward primer) and 5′-CATACTTAATATTATACTTGTCATTATCTGCGTCGTTGGAGTCG-3′ (reverse primer). pGEMII18-E6153PKA contains one nucleotide substitution (G^562^→T) within the E6 sequence and replaces amino acid arginine 153 with leucine. The primer set used was 5′-CTCCAACGACTCAGAGAAACACAAGTATAA-3′ (forward primer) and 5′-TTATACTTGTGTTTCTCTGAGTCGTTGGAG-3′ (reverse primer). Bidirectional DNA sequencing of the complete HPV18 genomes confirmed insertion of the mutations and the absence of mutations outside these nucleotide positions.

Newborn foreskin circumcision tissue samples were collected from patients attending a general practitioner's practice with informed written parental consent (ethical approval no. 06/Q1702/45). Primary HFK were isolated from the foreskin tissue and maintained in SFM keratinocyte growth media (Invitrogen, Paisley, Scotland, United Kingdom) as previously described (20). Low-passage-number J2-3T3 fibroblasts were grown in Dulbecco's modified Eagle's medium (Invitrogen) supplemented with 10% newborn calf serum and 4 mM glutamine.

The generation of HPV18 genome-containing cell lines has been described previously (21). Briefly, pGEMII18-WT, pGEMII18-E6153PKA, and pGEMII18-E6ΔPDZ were digested with EcoRI to release the viral genomes and recircularized in the presence of T4 DNA ligase, and each was cotransfected with a plasmid carrying the neomycin resistance gene into second- or third-passage HFK using FuGENE 6 transfection reagent (Roche, Welwyn Garden City, United Kingdom). One day after transfection, cells were seeded onto feeder layers of γ-irradiated J2-3T3 cells and grown for 8 days in the presence of G418 antibiotic (PAA Laboratories, Yeovil, United Kingdom) in E medium containing epidermal growth factor (BD Biosciences, Oxford, United Kingdom) and fetal calf serum (HyClone, Thermo Fisher Scientific, Cramlington, United Kingdom). Emerging cell colonies were pooled and expanded on γ-irradiated J2-3T3 cells. The pGEMII18-WT, pGEMII18-E6153PKA, and pGEMII18-E6ΔPDZ genomes were transfected into HFK isolated from a minimum of three different donors. Once cell lines were established, total DNA was extracted and the presence of the appropriate mutation was confirmed by PCR and DNA sequencing.

### Southern analysis.

HPV18-containing keratinocytes were grown as monolayer cell cultures on γ-irradiated J2-3T3 cells and harvested once 70 to 80% confluence was reached. Total DNA was extracted from the cells as described previously and analyzed by Southern blotting using an [α-^32^P]dCTP-labeled HPV18 genomic probe (21).

### Organotypic raft culture, chromogenic *in situ* hybridization (C-ISH), and immunocytochemistry.

Organotypic raft cultures were prepared as previously described (22). Each cell line was grown in an organotypic raft culture at least three times and harvested after 13 days of growth. To detect cells undergoing cellular DNA synthesis, the synthetic thymidine analogue bromodeoxyuridine (BrdU) (Sigma-Aldrich, Poole, United Kingdom) was added to the medium to a final concentration of 20 μM for 14 h prior to harvest. Organotypic rafts were fixed in formaldehyde and paraffin embedded, and 4-μm sections were taken (Propath UK, Ltd., Hereford, United Kingdom). Sections were treated with hematoxylin and eosin stain to observe morphology.

For immunohistochemistry, sections were first subjected to low-temperature antigen retrieval, as described elsewhere (23). For detection of incorporated BrdU, sections were stained with a fluorescein isothiocyanate (FITC)-conjugated antibody specific to BrdU (BD Biosciences, Oxford, United Kingdom). Cyclin B1 was detected using the antibody H-433 (Santa Cruz Biotechnology, Inc.), HPV18 E4 was detected with a rabbit polyclonal antibody that was raised against a glutathione *S*-transferase HPV18 E1^E4 fusion protein (21), and L1 was localized in organotypic raft sections by using a mouse monoclonal antibody (clone 5A3; NovaCastra Laboratories, Newcastle upon Tyne, United Kingdom). Immune complexes were detected using antispecies antibodies conjugated to Alexa Fluor 488 or Alexa Fluor 594 (Invitrogen). Nuclei were visualized using the DNA stain 4′,6-diamidino-2-phenylindole (DAPI).

To detect nuclei positive for viral DNA amplification, sections were probed with biotin-conjugated HPV DNA probe specific for high-risk HPV types, using Leica Bond-Max technology, as described by the manufacturer (Leica Microsystems, Milton Keynes, United Kingdom).

### Western blotting.

Cells grown in monolayer cell culture were lysed in a small volume of 50 mM HEPES (pH 7.0), 250 mM NaCl, 0.1% NP-40 supplemented with Complete protease inhibitor cocktail (Roche), sonicated briefly on ice prior to clearing the lysate by centrifugation at 16,100 × *g* at 4°C for 15 min. For preparation of cell lysates from organotypic raft cultures, the raft was scraped carefully from the fibroblast-embedded collagen base and homogenized in 1 ml of ice-cold lysis buffer (50 mM Tris-HCl [pH 7.4], 150 mM NaCl, 1 mM EDTA, 1% [vol/vol] NP-40 supplemented with Complete protease inhibitor cocktail [Roche]) in a glass Dounce homogenizer. Following incubation on ice for 30 min, the lysate was cleared by centrifugation (16,100 × *g*) for 15 min at 4°C.

Equal amounts of protein were separated by SDS-polyacrylamide (10%) gel electrophoresis (Tris-glycine), and resolved proteins were transferred to nitrocellulose membrane. The HPV18 E6 protein was detected using the monoclonal antibody AVC399, kindly donated by Arbor Vita Corporation (Fremont, CA), and used as previously described (24), or monoclonal antibody G-7 (Santa Cruz Biotechnology, Inc.). HPV18 E7 was detected using a mouse monoclonal antibody (8E2; Abcam, Cambridge, United Kingdom). The rabbit anti-phospho-E6 antibody was generated and used as described previously (19). Protein loading was assessed by detection of GAPDH (glyceraldehyde-3-phosphate dehydrogenase) using a mouse monoclonal antibody purchased from Santa Cruz Biotechnology or by detection of β-actin (Sigma-Aldrich). The signals were developed using enhanced chemiluminescence (GE Healthcare UK, Ltd.) and detected by exposure to X-ray film or using the Fusion FX7 image acquisition system (Vilber Lourmat, France).

### *In vivo* degradation assays.

Two micrograms of plasmids expressing hemagglutinin (HA)-DLG1, Flag-MAGI-1, HA-SCRIB, or p53 was cotransfected with 4 μg of plasmids expressing the various HPV18 E6 proteins or the empty vector into HEK293 or Saos-2 (for p53 only) cells using Lipofectamine 2000. Cells were harvested after 24 and 48 h and lysed on ice in urea-containing buffer (9 M urea, 25 mM Tris-HCl [pH 7.4], 150 mM NaCl, supplemented with Complete protease inhibitor cocktail [Roche]). After brief sonication, the protein lysates were cleared by centrifugation (16,100 × *g*) for 15 min at 4°C. Equal amounts of the cleared lysate were resolved by SDS-PAGE and after transfer to nitrocellulose immunoblotted with anti-HA (Covance), anti-Flag (Sigma-Aldrich), or anti-p53 (DO-1) (a kind gift from Roger Grand, University of Birmingham) antibodies. The transfection efficiencies of the different plasmids were assessed by cotransfection of a green fluorescent protein (GFP)-expressing plasmid.

### Cell growth assays.

Cells were seeded at a density of 4 × 10^4^ onto a layer of γ-irradiated J2-3T3 cells in six-well plates. Growth of the cells was monitored for up to 6 days by harvesting and counting the viable cells following removal of fibroblast feeder cells. Cell-doubling times for the various cell lines were calculated (http://www.doubling-time.com/compute.php). To monitor the effects of chemical activators of the cAMP-dependent PKA pathway on cell growth, forskolin (FK) and 3-isobutyl-1-methylxanthine (IBMX; Sigma-Aldrich) were each solubilized in dimethyl sulfoxide (DMSO) and used in combination at concentrations of 50 μM and 500 μM, respectively; dibutyryl-cAMP (Bt_2_-cAMP) (Biolog, Hayward, CA) was prepared in Hanks' balanced salt solution at 1 mM. The modifiers of PKA signaling were added to cell cultures 2 days after seeding. Cells were harvested following removal of feeder fibroblasts, and viable cells were counted at 12 and 48 h postaddition of the drug. Equivalent amounts of DMSO were added as a vehicle control for FK/IBMX-treated cell cultures. Ten separate cell counts were taken for each time point, and data were collected from three independent experiments. Cells treated with FK and IBMX were also harvested after 2 h of treatment and lysed in E1A buffer (250 mM NaCl, 0.1% NP-40, 50 mM HEPES [pH 7.0]) supplemented with the Complete protease inhibitor cocktail (Roche) and phosphatase inhibitor cocktail III (Sigma-Aldrich). The prepared lysates were analyzed by Western blotting for expression of phosphorylated E6 protein.

## RESULTS

### The stable maintenance of viral episomes in primary keratinocytes is affected by deletion of the E6 PBM, but not by disruption of the PKA recognition motif.

**Fig 1:**
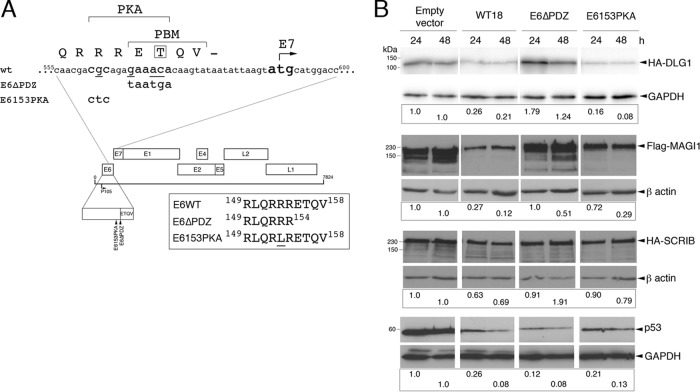
Details of E6 C-terminal mutations in HPV18 genomes and analysis of the activity of these mutations in an *in vivo* degradation assay. (A) Two mutant HPV18 genomes were constructed. E6ΔPDZ contains base substitutions at nucleotides 567, 570, and 571, thereby altering the codons for glutamic acid 155 and threonine 156 to translation termination codons. This mutant genome supports expression of an E6 protein in which the PBM, ETQV, is deleted. E6153PKA contains a single base substitution at nucleotide 562 that alters the codon for arginine 153 to leucine; the mutant protein expressed from this genome is not a substrate for PKA and targets cellular PDZ proteins in a constitutive, PKA-independent manner (17). The position of the initiator methionine codon for the E7 protein, which is separated from the translation termination codon of E6 by 8 nucleotides, is indicated. (B) *In vivo* E6-mediated degradation assays of epitope-tagged PDZ domain-containing targets (DLG1, MAGI-1, and SCRIB) and p53. Urea-soluble cell extracts were harvested at 24 and 48 h posttransfection, and the level of E6 targets was assessed by Western blotting. Each antibody data set (all four transfections) was taken from the same exposure. Shown below each degradation experiment are the amounts of PDZ proteins or p53 remaining relative to the transfections with empty vector (following normalization to the loading control [GAPDH or β-actin]). The efficiency of transfections of each set of expression plasmids was monitored by inclusion of a GFP-expressing plasmid, and the efficiencies were shown to be equivalent (WT, 32%; E6153PKA, 33%; E6ΔPDZ, 32%) between the cotransfections.

To gain insight into the biological significance of the PKA recognition motif to E6 function in the HPV18 life cycle, two separate mutations were constructed within the E6 gene of the HPV18 genome (Fig. 1A). In the E6153PKA mutant genome, the essential arginine residue (R153) of the PKA consensus motif was changed to a leucine residue. In a previous study, it was shown that a leucine substitution of this arginine in HPV18 E6 was sufficient to abrogate PKA phosphorylation of E6 in cells (17). Since PKA-mediated phosphorylation of the threonine residue within the PBM could no longer occur, the mutant E6 protein degraded the PDZ target DLG1 in a constitutive, PKA-independent manner (17). In the second mutant genome (E6ΔPDZ), the four residues that constitute the PBM were deleted from the E6 protein by changing the glutamic acid and threonine codons at positions 155 and 156, respectively, to translation termination codons (Fig. 1A). The E6 protein expressed from this mutant genome is unable to target cellular PDZ substrates (11).

To confirm that the mutations behave as predicted toward PDZ protein substrates, the individual E6 mutant proteins were examined for their ability to target epitope-tagged PDZ proteins, DLG1, MAGI-1, and hSCRIB in *in vivo* degradation assays (Fig. 1B). In HEK293 cells, the E6153PKA mutant protein behaved as the wild-type E6 protein and targeted DLG1 and MAGI-1 for degradation, albeit showing, relative to the wild-type protein, a marginally greater efficiency in degrading DLG1 and a slightly lower efficiency in targeting MAGI-1 for degradation. The wild-type protein has only a weak effect upon hSCRIB since it is not a preferential PDZ target of the HPV18 protein (25), and likewise, there is little degradation of hSCRIB in the presence of E6153PKA. As expected, deletion of the E6 PBM abrogated degradation of the PDZ proteins, while neither mutation inhibited the ability of E6 to degrade p53 in Saos-2 cells (Fig. 1B). Together, these data indicate that the mutations in the PKA-PDZ module do not adversely alter substrate selectivity. Since there was only a slight enhancement in degradation of DLG1 following expression of the constitutively active mutant E6153PKA, the majority of the wild-type E6 protein is likely to be in an unphosphorylated state with regard to the PBM and thus active toward degradation of its PDZ domain-containing targets (19). However, we cannot exclude the possibility that mutation of arginine 153, a residue not essential for binding to PDZ domain-containing substrates, might have a subtle effect on degradation of some targets, such as MAGI-1, that is irrespective of the phosphorylation status of the protein (26).

**Fig 2:**
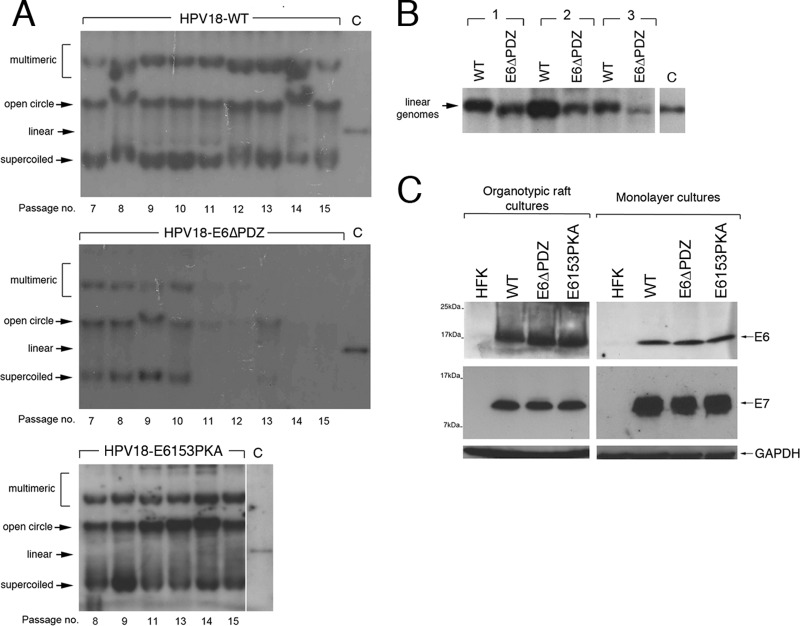
Stable maintenance of HPV18 episomes is dependent on E6 PBM function but independent of PKA recognition. (A) Southern analysis of equal amounts of total DNA extracted from HFK transfected with wild-type (HPV18-WT) or mutant (HPV18-E6ΔPDZ and HPV18-E6153PKA) genomes upon serial passaging of the cells. To assess for the presence of episomal forms (supercoils and open circles) of the viral genome, the DNA was restricted with the endonuclease BglII, which does not digest the HPV18 genome. To control for the presence of bacterially derived input DNA, the digests included the endonuclease DpnI. Linearized HPV18 genomes are shown as a copy number control (lane C [5 copies per cell]). (B) The E6ΔPDZ genomes establish at a lower copy number in HFK than the WT genomes, as shown by Southern analysis of total DNA taken from three different donors (passages P5 and P6) and digested with EcoRI, which linearizes HPV18 episomes, and DpnI (lane C, 5 copies per cell). (C) Detection of E6 and E7 proteins in equal amounts of NP-40 detergent-soluble protein lysates prepared from cells grown in monolayer cultures or from organotypic raft cultures. Levels of GAPDH were used as a loading control.

HFK were transfected with the wild type HPV18 genome, or each of the mutant genomes, E6ΔPDZ and E6153PKA, and stable lines were established. Southern blotting of total genomic DNA isolated from undifferentiated monolayer cell cultures showed that the wild-type and mutant viral genomes were established as extrachromosomal episomes (Fig. 2A). Generally, the E6ΔPDZ genomes were established as replicating episomes at a lower copy number than the wild-type or E6153PKA genomes, and further significant reduction in the level of the E6ΔPDZ episomes occurred upon extended passage of the cells (Fig. 2A and B). The marked decrease in the number of E6ΔPDZ episomes was accompanied by viral DNA integration, as assessed by disruption of the E2 gene (27) (data not shown). The sharp reduction in the maintenance of E6ΔPDZ genomes was observed in four out of five different donor backgrounds; in three of these, the reduction occurred by passage 11 (equivalent to ∼44 to 46 cell population doublings), while in another, they occurred after ∼76 cell population doublings (passage 18). However, in a fifth donor, the level of E6ΔPDZ genomes was maintained at a copy number equivalent to that of the wild-type episomes even after 108 to 110 population doublings (passage 27). Reduction in wild-type episomes was also observed in one of the donor backgrounds, but decreased viral genome maintenance occurred at a later passage (passage 18; ∼72 population doublings) than in the E6ΔPDZ genome-containing cells (passage 11; ∼46 population doublings [data not shown]).

These data indicate that HPV18 E6 PBM function is necessary for the efficient establishment of viral episomes and for maintenance of genome replication in primary keratinocytes, findings that concur with similar mutational studies of the E6 PBM of HPV16 and HPV31 in immortalized and primary keratinocytes, respectively (6, 7), although our data indicate that persistence of the mutant genomes is influenced by the donor background. In addition, the stable maintenance of the E6153PKA episomes, even after extended passaging of the cells, suggests that the loss of PKA regulation of the E6 C-terminal PBM does not adversely affect long-term maintenance of replication of the HPV18 genome (Fig. 2A).

In order to ensure that the mutations do not adversely affect the levels of E6 and/or E7 expression, the steady-state protein expression levels of the two viral oncoproteins were assessed in lysates prepared from cells grown in monolayer culture and following stratification in organotypic raft culture for 13 days (Fig. 2C). Western blotting of equivalent amounts of protein showed little variation in the expression levels of both oncoproteins between the various cell lines in undifferentiated cells or in the stratified cultures. These results indicate that the nucleotide changes within the E6 gene do not adversely affect E7 protein production and the alteration in PBM activity is not associated with any change in the steady-state expression of the E6 protein in primary keratinocytes.

### The integrity of the E6 PBM is critical for differentiation-dependent HPV18 vegetative functions in primary keratinocytes.

**Fig 3:**
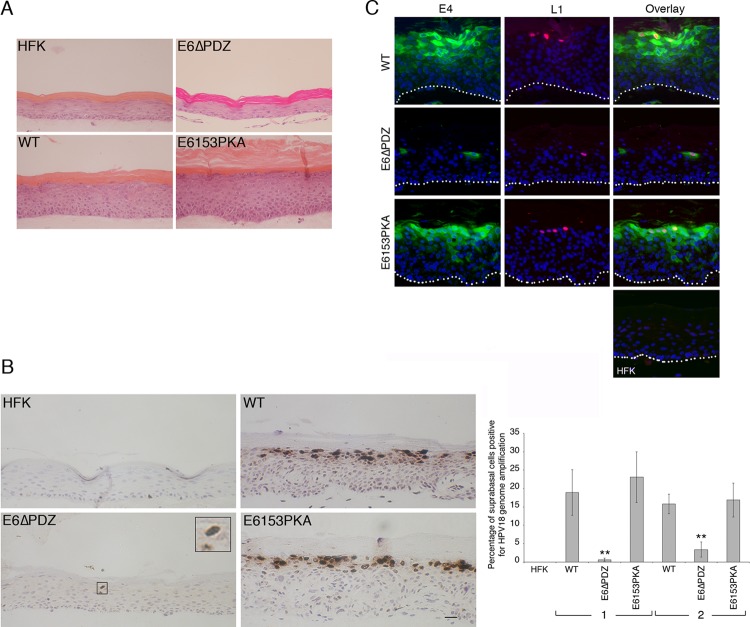
E6 PBM function is required for viral genome amplification and expression of E4 and L1 in suprabasal keratinocytes. (A) To show the morphological detail of the stratified structures, sections of organotypic rafts were stained with hematoxylin and eosin. It is important to note that for organotypic raft culture of E6ΔPDZ cells, it was ensured that cell passages prior to loss of viral episomes were used. (B) Nuclei positive for HPV18 DNA amplification (brown staining) were identified by chromogenic ISH. Viral DNA-positive nuclei were rare in sections of E6ΔPDZ rafts, with an example shown enlarged in the inset. Scale bar, 25 μm. The graph shows the percentage of suprabasal cells containing HPV 18 DNA-positive nuclei for HFK, WT, E6ΔPDZ, and E6153PKA genome-containing cells in different donor backgrounds (brackets 1 and 2). The data, shown as means with standard deviations (**, *P* < 0.01, Student's *t* test), were derived from five fields of view of each section of raft, with between 450 and 1,350 cells counted for each raft section. (C) Sections of rafts stained for E4 (green) and L1 (red) and nuclei visualized using DAPI staining (blue). Suprabasal cells of raft cultures of WT and E6153PKA genomes showed areas of E4 and L1 staining, but E4- and L1-positive cells were rarely observed in rafts of E6ΔPDZ genome-containing cells. No E4 or L1 staining occurred in raft cultures of donor keratinocytes (HFK). White dotted lines indicate the basal cell layer.

To investigate the effect of changes to E6 PBM function on epithelial stratification and the productive virus life cycle, organotypic raft cultures of the various cell lines were grown, and paraffin-embedded formalin-fixed sections were stained with hematoxylin and eosin (Fig. 3A). In comparison to structures produced from normal primary keratinocytes, the presence of the wild-type HPV18 genomes was associated with a thickening of the parabasal and spinous cell layers and occurrence of areas where nuclei had been retained throughout all layers of the stratified structures, as previously shown (21, 22). Organotypic rafts produced from cells carrying the E6153PKA genomes had a similar morphology to the wild-type genome rafts, with the exception that they generally showed greater thickness of the nucleus-containing cell layers (Fig. 3A). In contrast to the morphology of the wild-type or E6153PKA rafts, the overall thickness of rafts produced from E6ΔPDZ cells was consistently reduced, and their appearance more closely resembled the morphology of the structures produced from the untransfected donor cells. A similar phenotype was also reported for HPV31 genomes that lacked the E6 PBM (6) and for HPV18 genomes that were unable to express a full-length E6 protein (10). Staining of the rafts with markers of epidermal differentiation showed that the pattern of expression of both early (differentiation-specific keratins) and late (involucrin and filaggrin) markers in the presence of the mutant genomes was similar to that of rafts containing the wild-type genomes (data not shown).

To determine if the changes in E6 PBM activity affected viral genome amplification, chromogenic *in situ* hybridization (C-ISH) using an HPV-specific probe was performed on organotypic raft sections. As shown in Fig. 3B, intensely stained nuclei indicating cells undergoing viral DNA amplification are prominent in cells of the upper suprabasal cell layers of rafts of cells carrying the wild-type genomes, but not in rafts of the untransfected donor keratinocytes. A similar pattern of viral DNA amplification was also evident in E6153PKA rafts, but not in the E6ΔPDZ rafts, where HPV DNA-positive nuclei were rarely observed. Quantification of the data in this donor keratinocyte background showed a significant reduction (*P* < 0.005) in the number of cells undergoing viral genome amplification in the absence of a functional E6 PBM, an observation that was consistent across the different donors (Fig. 3B). For the generation of the E6ΔPDZ rafts, cells were used prior to loss of detection of episomes. Since the steady-state levels of E6 and E7 protein expression between the wild-type and mutant genomes were equivalent (Fig. 2C), this confirmed the presence of the genomes in these cells.

Expression of the viral capsid proteins occurs in the more differentiated cells, so to determine if changes in E6 PBM activity affect capsid production, rafts were stained with an antibody that recognizes the major capsid protein L1 (Fig. 3C). Sections were costained for the E4 protein, since the function of this protein has been shown to be necessary for efficient genome amplification and late viral gene expression (21, 28, 29). In rafts produced from cells containing viral genomes that retain a functional E6 PBM (WT and E6153PKA), abundant E4 staining occurred in the cytoplasm of cells of the mid and upper suprabasal cell layers, and a proportion of the nuclei in these cells were dual stained for the L1 protein (Fig. 3C). However, in E6ΔPDZ raft sections, only an occasional suprabasal cell was positive for E4 and L1 protein expression (Fig. 3C). Taken together, these data indicate that disruption of the PKA recognition motif at the C terminus of E6 does not adversely affect the ability of the mutant genomes to support vegetative functions following stratification in organotypic raft culture and therefore suggest that a constitutively active E6 PBM is not detrimental to the vegetative cycle. However, loss of E6 PBM function severely affects the vegetative cycle, with a significant reduction in the number of cells that support viral DNA amplification and the expression of late viral proteins, suggesting that targeting of cellular PDZ proteins is required for efficient viral replication in suprabasal keratinocytes.

### Changes in E6 PBM function affect S-phase activity in the basal and suprabasal compartments of organotypic raft cultures.

**Fig 4:**
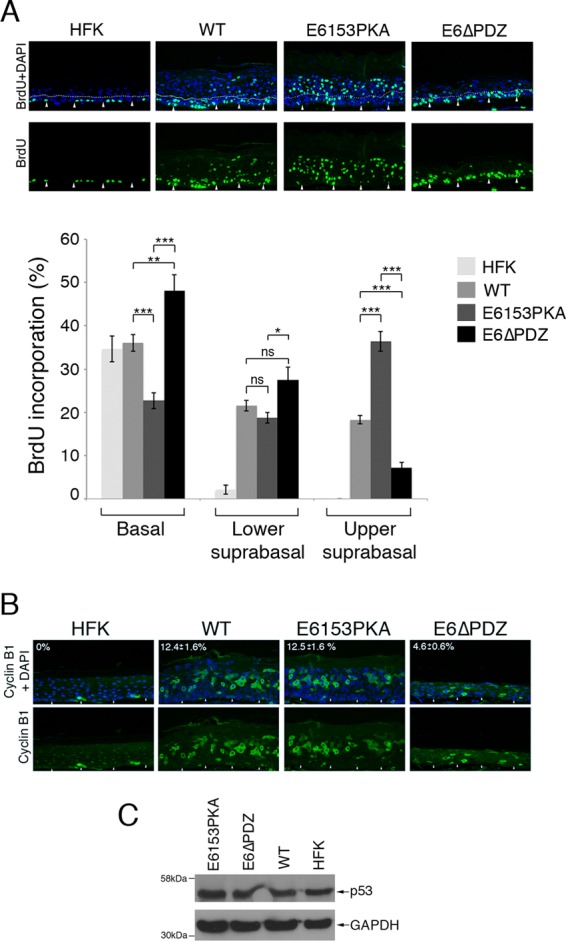
Changes in basal and suprabasal cellular DNA synthesis correlate with the activity of the E6 PBM. (A) Organotypic raft cultures were incubated with BrdU to identify nuclei positive for cellular DNA synthesis. Immunofluorescence staining was performed with an anti-BrdU antibody (green), and nuclei were visualized using DAPI staining (blue). The arrowheads identify the basal cell layer, and the dotted line differentiates the lower and suprbasal cell layers. The bar graph shows the percentage of basal, lower suprabasal (parabasal and lower spinous), and upper suprabasal (upper spinous and granular layers) nuclei positive for BrdU incorporation, which was calculated from multiple (between 4 and 6) fields of view of raft sections using a 20× objective. The data, shown as the means ± standard errors, are from four different donor lines. Statistical analysis was performed using Student's *t* test. (B) Sections of organotypic rafts stained with an anti-cyclin B1 antibody (green) and counterstained with DAPI (blue). The percentage of suprabasal cells positive for cytoplasmic cyclin B1 expression, calculated from 10 fields of view and from three different donor lines, is shown as the mean ± standard error. The paired Student's *t* test showed that the difference between E6ΔPDZ and WT rafts was significant (*P* > 0.0001). Arrowheads indicate the basal cell layer. (C) Western blot analysis of p53 protein expression in NP-40-containing lysates prepared from organotypic raft cultures showed no marked changes in p53 protein levels between the different mutants (E6ΔPDZ and E6153PKA) and the cells containing wild-type (WT) genomes. Levels of GAPDH were used to control for protein loading. HFK, untransfected primary keratinocytes.

Amplification of HPV genomes in postmitotic differentiated cells is dependent on reactivation of the host's cellular DNA replication machinery. This is largely mediated by the ability of E7 to stimulate S-phase reentry of these cells, although this activity can also be augmented by E6 (30, 31). To confirm that a suprabasal S-phase milieu is promoted in the presence of the mutant E6 proteins, organotypic raft cultures were incubated with the thymidine analogue bromodeoxyuridine (BrdU) 14 h prior to harvest, and sections were stained with an anti-BrdU monoclonal antibody to identify cells undergoing cellular DNA synthesis (Fig. 4A). Replication of the host chromosome is restricted to cells of the basal cell layer in raft cultures of the untransfected HFK, while in wild-type and mutant genome-containing raft cultures, BrdU-positive nuclei were detected in basal and suprabasal epithelial compartments, indicating that the mutant genomes support S-phase reentry of the differentiating cells. Interestingly, quantification of the number of BrdU-positive nuclei within the different epithelial layers of the raft revealed significant differences between the mutants and the wild-type genome-containing cells (Fig. 4A). While the numbers of BrdU-positive cells confined to the basal layers did not vary significantly between the untransfected HFK and wild-type genome-containing rafts (∼35% versus ∼36%), there was a decrease in the S-phase population in E6153PKA rafts (∼23%), and in E6ΔPDZ rafts, this population increased (∼48%). Within the parabasal and lower spinous layers (referred to as lower suprabasal) of the wild-type and both mutant rafts, the numbers of BrdU-positive cells were comparable (WT, ∼21%; E6153PKA, ∼19%; E6ΔPDZ, ∼27%). However, significant differences in the S-phase active population were observed in the upper suprabasal compartment (consisting of the upper spinous and granular layers); in comparison to wild-type genomes (∼18%), BrdU-positive nuclei were greatly increased in E6153PKA rafts (∼36%) but markedly decreased in the presence of the E6ΔPDZ genomes (∼7%). The data shown were derived from four different donor backgrounds (Fig. 4A).

These data indicate that the loss of PDZ targeting by E6 does not prevent cells from entering S phase as they leave the basal layer and begin to migrate upwards, but the PBM is detrimental to the maintenance of S-phase competent cells in the upper suprabasal layers. This is a likely explanation for the marked decrease in cells competent for viral genome amplification. High-level synthesis of the viral genome occurs in suprabasal cells that have moved from S phase into G_2_, and these G_2_-arrested cells are identified by accumulation of cytoplasmic cyclin B1. We observe a significant decrease of 63% (combined data taken from three donor backgrounds; *P* > 0.0001) in this population of G_2_-arrested cells following staining of the E6ΔPDZ rafts with a cyclin B1 antibody, relative to the wild-type genome-containing rafts, further illustrating the failure of E6ΔPDZ cells to maintain cell cycle activity in the upper cell layers (Fig. 4B).

Recent studies suggest that elevated levels of p53, induced in response to the activity of E7 in suprabasal keratinocytes, suppress viral DNA amplification. High-risk E6 proteins negate this host response by destabilizing p53. Deletion of the E6 PBM does not affect the ability of E6 to destabilize p53 *in vivo* (32) (Fig. 1B), and furthermore, we observed no increase of p53 protein expression in E6ΔPDZ differentiated cells compared to cells carrying the wild-type genomes (Fig. 4C).

In contrast to the E6ΔPDZ mutation, disruption of the PKA recognition motif that is critical for the phosphorylation of the PBM domain is associated with a marked increase in S-phase competent cells in the upper suprabasal cell layers, although this does not correlate with an increase in the number of cells amplifying their genomes relative to the wild-type genome-containing rafts (Fig. 3B). Indeed, there is no increase in the number of G_2_-arrested cells in the E6153PKA rafts upon comparison with those formed from cells carrying the wild-type HPV18 genomes (Fig. 4B).

### Disruption of the PKA recognition motif of E6 leads to an increase in the rate of growth of HPV18 genome-containing cells.

**Fig 5:**
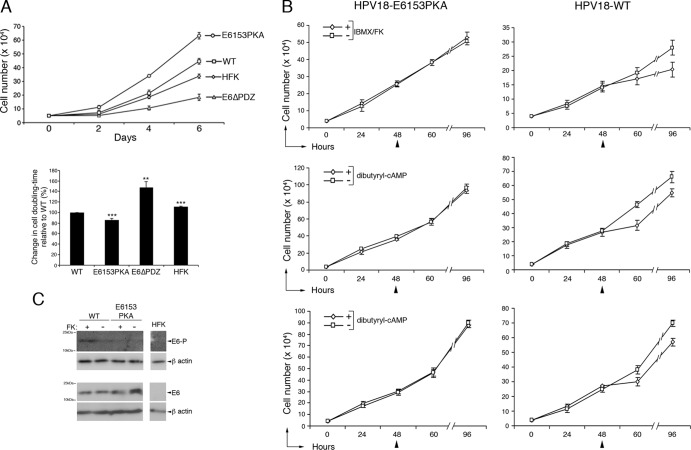
Activation of PKA signaling in HPV18 genome-containing cells attenuates cell growth. (A) Cell growth profiles of untransfected keratinocytes (HFK) and of HPV18 genome-containing cells generated in three donor backgrounds. Growth profiles were performed in duplicate for each donor and performed as three independent experiments. Bar graph, change in cell doubling time of the mutant genome-containing cell lines (E6ΔPDZ and E6153PKA) and the untransfected cells (HFK) relative to cells carrying wild-type genomes (WT). Data are shown as means ± standard errors, and statistical analysis was performed using Student's *t* test. (B) Growth assays of HPV18 genome-containing cells (WT or E6153PKA) treated with PKA activators (+) or vehicle (−) 48 h postseeding (arrowheads). Viable cells were counted at 12 and 48 h post-drug treatment, after removal of fibroblast feeder cells. Each data point (± standard deviation) shows data collected from 10 separate counts for each time point and from triplicate experiments. The data from two separate donor backgrounds are shown for treatment with dibutyryl cAMP. HPV18-WT (IBMX/FK), 72 h, *P* < 0.01, 96 h, *P* < 0.001; HPV18-WT (dibutyryl-cAMP), donor 1, 72 h, *P* < 0.001, 96 h, *P* < 0.01; donor 2, 72 h, *P* < 0.5, 96 h, *P* < 0.01. (C) Detection of phosphorylated E6 (E6-P) and total E6 (E6) in equal amounts (30 μg) of cell lysates prepared from WT or E6153PKA genome-containing cells treated with FK/IBMX (+) or vehicle (−). HFK cell lysates were included to assess for specificity of the antibodies, and levels of protein loading were assessed using β-actin.

The above results indicate that the E6153PKA genome-containing cells exhibit more of a hyperproliferative phenotype than cells containing the wild-type genome following organotypic raft culture (Fig. 3 and 4). To determine whether the growth of these cells was also affected in monolayer cell culture, cells were grown on γ-irradiated J2-3T3 fibroblasts and harvested at 2, 4, and 6 days after seeding. Viable cells were counted after removal of fibroblasts, and the combined analysis of the growth rates of cells from all three donor backgrounds is shown in Fig. 5A. In all of the different donor backgrounds, as expected, the wild-type HPV18 genome-containing cells grew at a higher rate than the untransfected donor primary keratinocytes (HFK). Deletion of the E6 PBM was associated with a marked reduction in the rate of cell growth with a significant increase in the doubling times of E6ΔPDZ cells compared to the wild-type genome-containing cells (Fig. 5A), a finding that concurs with a similar E6 deletion in the HPV31 genome (6). Moreover, as was also noted in the study of the HPV31 genomes, the growth of cells containing viral genomes lacking an E6 PBM is slower than the parent keratinocytes (Fig. 5A). In contrast, those cells carrying the E6153PKA genomes grew faster than the wild-type genome-containing cells, with a significant decrease in their doubling times. These data indicate that a function of the HPV18 E6 PBM is to facilitate efficient growth of primary keratinocytes and suggest that the conditional regulation of the E6 PBM by a cellular kinase has a role to play in controlling the growth of the viral genome-containing keratinocytes.

### Activation of PKA signaling in HPV18 genome-containing cells attenuates cell growth.

In light of our findings, the effect of increasing PKA activity on the growth of cells containing the wild-type HPV18 genome or the E6153PKA genome was investigated. PKA activity was stimulated by increasing intracellular cAMP levels, either by the addition of forskolin (FK) and 3-isobutyl-1-methylxanthine (IBMX) in combination or the cell-permeable PKA activator, dibutyryl-cAMP (Bt_2_-cAMP) to the monolayer cell cultures. Cells were seeded onto γ-irradiated J2-3T3 feeder cells, the chemical activators were added after 48 h, and viable cells were counted subsequently at 12 and 48 h. Where appropriate, identical amounts of vehicle were added to parallel cell cultures. In the presence of FK/IBMX, the growth of wild-type genome-containing cells was significantly repressed at both time points in comparison to the untreated cells, a consistent effect between different donors (Fig. 5B). In contrast, there was no significant change in the growth profile of E6153PKA genome-containing cells following addition of the PKA activators. A similar profile of growth response was also observed following the addition of Bt_2_-cAMP (Fig. 5B). These data indicate that only the growth of cells containing an HPV18 genome with an E6 PBM that is amenable to regulation by PKA is decreased in response to increased PKA activity. To determine if stimulation of PKA signaling is associated with phosphorylation of E6 in the genome-containing cells, lysates were prepared from cells treated with FK and IBMX in combination or by vehicle alone. Equal amounts of the lysates were analyzed by Western blotting using an antibody that detects total E6 and an anti-phospho E6 antibody that recognizes the phosphorylated HPV18 E6 PBM (19). The Western blot (Fig. 5C) shows that in the absence of PKA stimulation, E6 expressed from the wild-type genomes appears to be only weakly phosphorylated. However, increased E6 phosphorylation is observed following incubation of cells with FK and IBMX (Fig. 5C). While E6 could be detected in the E6153PKA genome-containing cells, there was no indication that it was phosphorylated in the absence or presence of PKA activators. These data suggest that E6 expressed in wild-type genome-containing cells is susceptible to PKA phosphorylation within the PBM and that this phosphorylation is dependent on the integrity of the overlapping PKA recognition site.

## DISCUSSION

Disruption of the integrity of the E6 PKA-PBM module in the complete HPV18 genome has profound effects upon keratinocyte growth and upon the viral life cycle. In a submerged monolayer culture, this effect is linked to PKA signaling since the growth of cells carrying the wild-type genomes decreases upon PKA activation and this is associated with increased phosphorylation of the E6 PBM. Loss of phospho-regulation, which renders E6 constitutively active for PDZ binding, is also associated with a more hyperplastic phenotype following stratification of the cells in an organotypic raft culture. The stratified structures are thicker than those produced from cells carrying the wild-type genomes, and there is a significant increase in the percentage of S-phase competent cells in the upper suprabasal cell layers (cells of the upper spinous layers and granular layers). This is in contrast to the morphology of rafts produced by cells containing genomes in which the E6 PBM has been deleted. These cells formed much thinner stratified structures, resembling those produced from the untransfected parent primary keratinocytes. In addition, there was a significant decrease in the percentage of S-phase cells in the upper suprabasal cell layers. These findings are consistent with studies in mice that showed that the E6 PBM was required for the hyperproliferative growth of mouse epithelium (32, 33). However, the numbers of BrdU-positive cells in the lower suprabasal layers, consisting of cells of the parabasal and lower spinous layers, were no different between the wild-type and mutant genomes. This is not surprising since reactivation of S-phase functions in cells as they migrate up from the basal cell layer is largely mediated by E7 (30). Taken together, our data indicate that the E6 PBM plays a critical role in the expansion of the S-phase competent cell population in the more differentiated cell layers. Failure to maintain this cell population is a likely explanation for the inability of the E6ΔPDZ mutant genomes to support viral DNA amplification in the upper suprabasal layers. Indeed, we observed fewer cells arrested in G_2_/M in these layers, the point in the cell cycle in which viral DNA amplification is thought to occur (10). While the mechanics of E6 PBM action on the expansion of these postmitotic cells are not yet clear, it is intriguing that the mutation of known E6 PDZ domain-containing targets, DLG1 and Scribble in Drosophila, leads to hyperplastic overgrowth and loss of cell polarity of epithelial cells (34, 35).

The morphology of the organotypic rafts produced from the E6ΔPDZ mutation is very similar to that observed in primary cells containing HPV18 genomes that are unable to synthesize full-length E6 (10, 36). Restoration of a wild-type morphology occurred upon ectopic expression of HPV18 E6 in *trans* but interestingly also with mutant proteins defective in p53 targeting, indicating that this activity of E6 is independent of p53 inactivation (36). In fact, although p53 is implicated in regulating viral DNA amplification, other cellular factors are likely to be involved, and these may be modulated by the E6 PBM function (36).

One other intriguing difference between the wild-type and mutant genomes was the level of S-phase activity in the basal layers of the rafts. Loss of PDZ domain targeting was associated with increased frequency of cells undergoing host cell DNA synthesis in this cellular compartment, while those cells containing genomes that have lost PKA regulation and have constitutively active PDZ binding potential were less proliferative. This observation was surprising in light of the growth behavior of these cells in submerged monolayer culture, where the opposite effects were obtained. However, monolayer cultures are nonpolarized cells undergoing symmetrical cell division, whereas in the rafts, the basal cells would exhibit apicobasal polarity and undergo asymmetrical as well as symmetrical division, cell processes that are regulated by some of the PDZ domain-containing targets of E6 (37, 38). Whether this phenotype is a reflection of the constitutive targeting of PDZ domain-containing substrates of E6 or a reflection of the inability of E6 to interact with potential phospho-dependent targets (such as 14-3-3 proteins) (19) remains to be determined.

Clearly, loss of PKA regulation of the E6 PBM does not interfere with the establishment or persistence of the HPV18 genomes. Deletion of the PBM, however, is linked to poor establishment of replication and a failure to maintain the viral episomes, a biology that is conserved between similar mutant genomes of other high-risk HPV types (6, 7). One would therefore predict that in these cells most of the E6 is likely to be unphosphorylated, since phosphorylation might be expected to mimic the E6ΔPDZ phenotype. Indeed, using phospho-specific antibodies directed against the HPV18 E6 phospho-PBM, very low levels of phosphorylated E6 are present in the monolayer cultures, while activation of PKA signaling induces a dramatic increase in the amount of phosphorylated E6. Furthermore, the steady-state levels of the wild-type and mutant E6 proteins are comparable in our HPV18 genome-containing cell lines, indicating that a change in E6 protein stability is an unlikely explanation for the phenotypes observed in primary keratinocytes.

How phosphorylation of the E6 PBM regulates the interaction with PDZ protein targets during the virus life cycle is far from clear. Structural studies of E6 PBM-PDZ complexes suggest that phosphorylation of the critical residue in the PBM would simply inhibit E6's ability to dock with PDZ domain-containing substrates (17–19). However, disentangling the biological effect of E6 PBM phosphorylation is complicated by the recent finding that phosphorylated E6 gains the ability to interact with cellular proteins that recognize this phosphorylated motif, such as 14-3-3 proteins (19). Thus, differential targeting of cellular proteins by E6 in the virus life cycle may be regulated by phosphorylation of E6 C-terminal sequences (39).

The evolutionary restriction of the E6 PBM to only those HPV types within the *Alphapapillomavirus* genus implicated as the etiological agents for the development of mucosal epithelial cancers suggests that the functions of the E6 PBM contribute to the oncogenic potential of these viruses, and indeed this is supported by both cell-based and animal studies (13, 32, 40–42). As reported here and elsewhere (6, 7), loss of E6 PBM function in the context of whole viral genomes is associated with viral DNA integration, a critical event in HPV carcinogenesis. On the other hand, abrogation of kinase recognition of the E6 PBM function is associated with increased cell growth of viral genome-containing cells and has also been associated with the enhancement of morphological transformation of keratinocytes (42). Therefore, it is reasonable to speculate that perturbation of the protein kinase signaling pathways that modulate E6 PBM function could lead to significant changes in E6 PBM function, which in turn could have a bearing on tumor promotion and progression.
